# Diagnostic Findings in a Confirmed Outbreak of *Brucella ovis* Infection in a Traditional Sheep Farm in Sicily (South-Italy)

**DOI:** 10.3390/pathogens10111472

**Published:** 2021-11-12

**Authors:** Paola Galluzzo, Sergio Migliore, Silvana Cascio, Santino Barreca, Marilena Alfano, Antonina Tagliarini, Anna Candela, Chiara Piraino, Lucia Galuppo, Lucia Condorelli, Hany A. Hussein, Manuela Tittarelli, Giuseppina Chiarenza

**Affiliations:** 1 Istituto Zooprofilattico Sperimentale della Sicilia “A. Mirri”, 90129 Palermo, Italy; paola.galluzzo@izssicilia.it (P.G.); santinobarreca@gmail.com (S.B.); marilena.alfano@izssicilia.it (M.A.); antonietta.tagliarini@izssicilia.it (A.T.); anna.candela@izssicilia.it (A.C.); chiara.piraino@izssicilia.it (C.P.); galuppolucia@gmail.com (L.G.); luciacond1980@gmail.com (L.C.); hany.ahmed@izssicilia.it (H.A.H.); giuseppina.chiarenza@izssicilia.it (G.C.); 2 Dipartimento Scienze e Tecnologie Biologiche Chimiche e Farmaceutiche, University of Palermo, 90133 Palermo, Italy; 3 Azienda Sanitaria Provinciale di Trapani, 91100 Trapani, Italy; silvanacascio@asptrapani.it; 4 Istituto Zooprofilattico Sperimentale dell’Abruzzo e del Molise “G. Caporale”, 64100 Teramo, Italy; m.tittarelli@izs.it

**Keywords:** *Brucella ovis*, ovine epididymitis, rams, ewes, diagnosis

## Abstract

Aim of this study is to report a laboratory investigation performed following the isolation of *Brucella ovis*, causing ovine epididymitis, in a traditional sheep farm in Sicily (South Italy). This disease represents a newly emerging risk for Italian livestock and is listed among diseases of EU priority (EU Reg 2016/429). Blood samples from 56 rams and 143 ewes were analyzed by both Enzyme-Linked Immunosorbent Assay (ELISA) and Complement Fixation Test (CFT). Genital swabs from all rams and 15 lactating ewes were collected to perform real-time PCR. Eighteen serologically positive rams were slaughtered and postmortem-inspected. Samples of testicle, epididymis, lymph nodes, and urine were also collected in order to perform microbiological, molecular, and histopathological analysis. Twelve slaughtered rams showed anatomo-pathological lesions. Real-time PCR for *B. ovis* BOV_A0504 gene was positive for 13 testicles and epididymis and 11 urine while *B. ovis* was isolated from epididymis and testicles of 7 slaughtered rams. This is the first exhaustive laboratory report of a microbiological, molecular, and serological pattern of the disease in sheep in Italy. Despite the impact on health and animal welfare, the epidemiology of *B. ovis* infection is still unknown, particularly in our country where the disease is considered endemic.

## 1. Introduction

*Brucella ovis* is a Gram-negative and naturally rough (R) bacterium belonging to the *Brucella* genus and is the causative agent of ovine epididymitis [1]. The disease affects sheep exclusively, causing genital lesions and overall reproductive failure. The main clinical manifestation in rams is epididymitis (either uni or bilateral), orchitis, and infertility [2]. Despite being nominated as ‘ovine epididymitis’, *B. ovis* infection can also induce clinical signs in ewes such as placentitis, abortion, and stillbirth. For this reason, the term ‘*Brucella ovis* infection’ is preferred, as epididymitis in rams can be caused by a large variety of pathogenic agents [2,3].

The disease is mainly transmitted via mating, ewes act as a passive reservoir. The occurrence of infection in a healthy farm is linked to the entry of infected animals: this negatively affects the fertility rate of the flock which might remain reduced even after eradication of the diseased animals [4]. Rams generally develop a subacute or chronic infection and shed *B. ovis* intermittently with semen, genital secretions, and urine for at least 2–4 years. Lesions were observed in 20–50% of naturally infected rams, while in artificially infected heads, the percentage of rams with lesions varies from 30% to 50% [2,3].

The prevalence and incidence in naïve sheep populations not subjected to prophylaxis are usually very high, especially when the disease is first reported in free regions, with 2–67% of rams and 9–50% of total animals being infected. Manifestation of clinical lesions in infected rams ranges from 20 up to 50% and abortion from 25 up to 60% of pregnant ewes [2,3,5].

*B. ovis* infection was reported in sheep farming worldwide but to date, the real distribution of *B. ovis* infection in the world is largely unknown (EFSA). Eradication programs/plans are not compulsory and successful conclusions of eradication campaigns have never been reported [3].

Unlike the other species of *Brucella*, *B. ovis* is not classified as a zoonosis and although it is considered to have a less severe health impact than the other species affecting ruminants, it is a cause of significant losses related to hypofertility and related concerns due to the impact on the genetic selection in the farm [6]. Recently, *B. ovis* infection has been assessed according to the criteria of the Animal Health Law (AHL), Regulation (EU) No 2016/429 and it was considered eligible to be listed (Commission Delegated Regulation (UE) 2018/1629) for control measures as laid down in Article 5 (3) of the AHL [7] (EU, 2021).

*B. ovis* infection doesn’t show peculiar, pathognomonic clinical symptoms and often the disease is subclinical and could circulate in the flock without suspicion. Several diagnostic tests are available today, including those recognized by OIE and EU regulations [8]. Other bacteria, such as *Actinobacillus seminis*, *Histophilusovis*, *Haemophilus* spp., *Corynebacterium pseudotuberculosis ovis*, *Chlamydophila abortus*, or *B. melitensis* are responsible for ovine epididymitis and more than 50% of the cases related to *B. ovis* infection normally do not show any visible lesion to the epididymis [9]: this makes it difficult to suspect just by clinical examination, the presence of the disease.

Diagnosis of *B. ovis* infection is based on clinical observation of genital organs, or with the help of laboratory tests such as antibodies detection through serological tests (CFT, ELISA, etc), bacterial isolation from semen and urines, or molecular tests (PCR) to detect DNA of the pathogen [10]. For certainty in the diagnosis, it is appropriate to repeat the sampling as the excretion is often intermittent. Although the Enzyme-Linked Immunosorbent Assay (ELISA) sensitivity is higher than Complement Fixation Test (CFT) the latter is the most widely used for *B. ovis* diagnosis due to its simplicity, cost, and lack of internationally recognized standardized ELISA assay. Therefore, CFT remains the most suitable test to certify individual animals before handling, even for international trade [9].

Despite the renewed attention of the European legislator on *B. ovis* infection, the information about the disease detection in the field remains scanty and to great extent incomplete, especially in traditional sheep farming where the infection is underestimated due to its subclinical occurrence.

In the present study, we described the diagnostic findings observed in an outbreak of *B. ovis* infection in a traditional sheep farm in Sicily in order to provide more details for rapid and proper disease detection.

## 2. Results

### 2.1. Serological Analysis

The serological diagnosis was carried out using both CFT and ELISA tests in order to assess the efficacy of these two methods and to have more data for proper evaluation of *B. ovis* infection in the examined farm. Anti-*B. ovis* antibodies were detected in 29 sera (51.8%) of rams by CFT assay whereas ELISA confirmed a larger number of samples, highlighting 35 rams (62.5%) positive to *B. ovis*.

**Figure 1:**
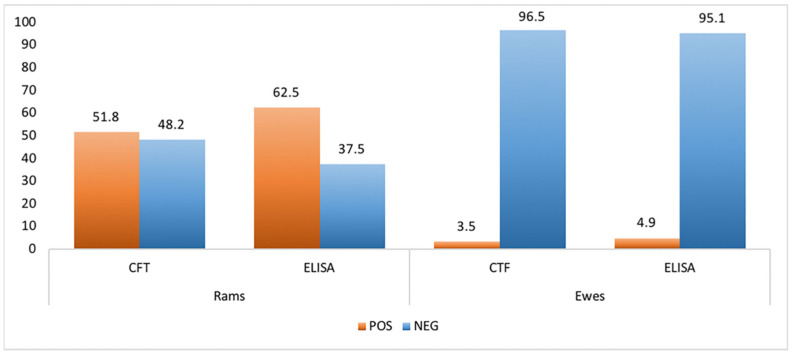
Serological results were obtained by Complement Fixation Test (CFT) and Enzyme-Linked Immuno Assay (ELISA) tests to evaluate the presence of anti-*B. ovis* antibodies in rams and ewes sera.

In contrast, the 143 sampled ewes showed a lower seroprevalence than rams with 5 (3.5%) and 7 (4.9%) heads positive to anti-*B. ovis* antibodies detected by CFT and ELISA, respectively (Figure 1). Despite ELISA appearing more sensitive, the two tests used revealed good agreement with a concordance of 88% and the K Cohen of 0.757.

### 2.2. Ante-Mortem and Post-Mortem Examination

**Figure 2:**
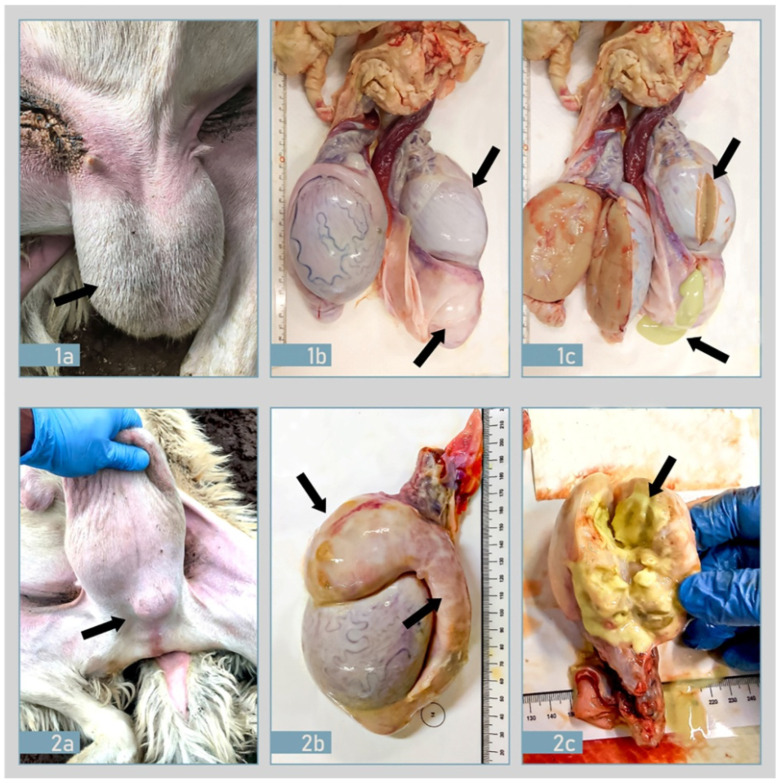
Anatomo-pathological findings in two different rams (**1**,**2**) naturally infected with *B. ovis*. (**1a**) Genital organs appearance with testicular asymmetry; (**1b**) Right testis showed atrophy with an absence of vascularization, diffuse fibrosis, and thickness of tunica vaginalis firmly adhered to the testis, and enlargement of the tail of epididymis in comparison with apparently normal left testis; (**1c**) Right testis atrophy with the tail of epididymis filled with yellowish caseous viscous fluid (purulent exudate). (**2a**) Subcutaneous mass on external examination; (**2b**) body and tail of left epididymis are severally increased in size with fibrosis/adherence of tunica vaginalis and epididymal abscess. (**2c**) Epididymis abscess: the body of epididymis is increased in size with a focal area in cut surface with yellowish caseous material.

Clinical examination and testicular palpation revealed the presence of specific lesions located in the epididymis and/or testicle. A total of 15 rams showed lesions (epididymitis and/or orchitis) and no other intercurrent pathologies were observed. In agreement with the owner and thanks to the support of the Veterinary Service of Trapani symptomatic and serological positive rams (18) were regularly slaughtered and inspected. Out of the total of 18 rams, 6 didn’t show lesions while the remaining 12 (66.67%), showed testicular asymmetry, hardness related to atrophy or hypertrophy, and epididymitis (Figure 2).

Anatomo-pathological changes were mainly identified in epididymitis, in the tunica vaginalis, and the parenchyma of the testicles: change varied from moderate to marked increase in the volume and modification in the consistency of the organ. The epididymal changes were generally unilateral, and the epididymal tail was more often affected than the head or the body (Figure 2).

### 2.3. Histopathological and Immunohistochemical Findings

**Figure 3:**
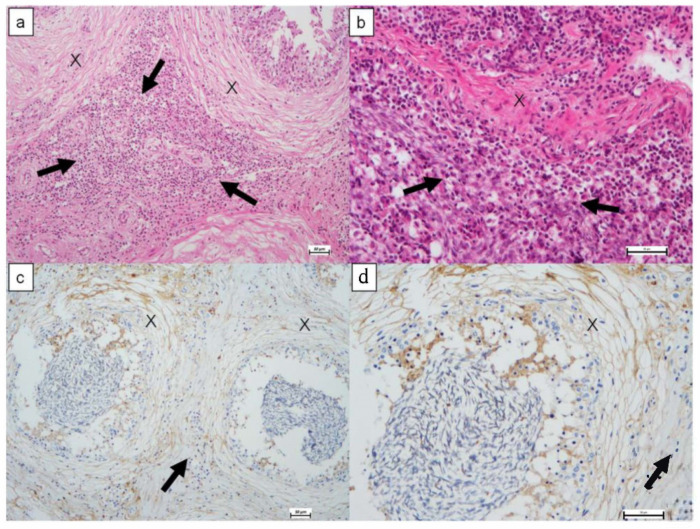
(**a**): Epididymis (X) with focal accumulations of lymphocytes, plasma cells, and neutrophils with necrotic debris (arrow); (**b**): surrounding interstitium characterized by mixed leukocytic infiltrate, predominantly macrophages (arrow), Haematoxylin-eosin stain, (**c**,**d**): epididymal ducts (X) containing *B. ovis* antigen (brown particles) and surrounded by a mantle of neutrophils, lymphocytes, plasma cells and macrophages (arrow). Both sections are stained for immunohistochemistry using anti–*B. ovis* antibody and hematoxylin counterstain. Bar size 50 µm.

Histological examination of the reproductive system tissues revealed evident epididymitis, ampollitis, and seminal vesiculitis on 11 rams (61.1%). The epididymitis showed focal pictures and could be classified as mild to severe. Histological lesions were characterized by mild to moderate focal accumulations of scattered lymphocytes and plasma cells or as perivascular sleeves in the interstitial connective tissue (Figure 3a,b). Focal accumulations of lymphocytes, plasma cells, and neutrophils with necrotic debris have also been observed in the lumen of the spermatic ducts. Accumulation of cellular debris, in some cases, resulted in the complete obstruction of the epididymis with subsequent abscess formation. Immunohistochemical staining revealed *B. ovis* bodies in the epididymal lesions. *Brucella*-specific staining was also detected within the cytoplasm of interstitial macrophages, epithelial ductal elements, macrophages, and neutrophils (Figure 3c,d). Positive cytoplasmic immunohistochemical staining was very intense in epithelial cells, especially lining the epididymal ducts; while extracellular brucellae were observed in the interstitium and spaces between the epididymal ducts.

### 2.4. Molecular Findings

Despite the official diagnosis in Italy only being provided for serological tests and clinical examination of animals, real-time PCR was performed on genital swabs and milk samples from living animals and tissues from slaughtered rams, in order to improve our data. The real-time PCR for *B. ovis* was based on a unique genetic locus, BOV_A0504, which was identified by in silico comparisons with other *Brucellae* and confirmed on several reference strains by Hinić and colleagues [11].

BOV_A0504 gene fragment for *B. ovis* was detected in 18 (32.1%) of the 56 preputial swabs.

Regarding samples collected from 15 dairy ewes, milk samples and vaginal swabs showed the presence of *B. ovis* target gene only in 6 out of 15 animals. Particularly positivity was detected in 6 (40%) and 3 (20%) of milk and vaginal swabs, respectively. With regard to molecular investigations carried out on tissue, a specific *B. ovis* gene fragment was confirmed in 13 (72.2%) testicles and epididymis and in 11 (61.1%) urine samples. Samples reported ct values ranging from 27.5 to 37.

### 2.5. Microbiological Findings

Testicles, epididymis, lymph nodes, and urine samples collected from the 18 slaughtered rams were subjected to microbiological investigation in order to isolate *B. ovis* strains. No *Brucella* spp. was isolated from lymph nodes and urine samples. Colonies attributable to *Brucella* spp. were identified in testes and epididymis of 7 animals (38.9%). Isolated bacterial colonies were subcultured onto blood agar plates where they appeared transparent, non-hemolytic, and rather small in size. On Gram staining, all isolated colonies appeared as small Gram-negative coccobacilli. At biochemical tests strains were negative for growth in MacConkey agar, growth in CO_2_ atmosphere, oxidase test, mobility, urease and H_2_S production, glucose oxide fermentation. Contrary, isolated strains tested were positive for catalase, growth in presence of 50 μg/mL both basic fuchsin and thionin. No agglutination was observed with *Brucella* A and M anti-serum and isolated strains didn’t grow during phages characterization. All tests led to strain identification such as *B. ovis*.

All isolated strains were confirmed as *B. ovis* by AMOS-PCR. The PCR provided the characteristic 976-bp amplicon for *B. ovis*.

The 7 rams from which *B. ovis* was isolated were also positive for antibodies screening, DNA detection, and showed evident pathological changes of the reproductive system.

## 3. Discussion

In this study, we showed for the first time in Italy, an exhaustive diagnostic report on *B. ovis* infection in a traditional Sicilian sheep farm. Despite the impact on health and animal welfare, information about the distribution and the diagnostic findings of *B. ovis* infection remains scarce and largely incomplete in most of the world, probably due to its non-pathogenicity to humans than other zoonotic *Brucella* spp. strains [12]. For this reason, we reported a complete clinical and laboratory profile of an outbreak identified in Sicily. Laboratory data relating to serological tests, isolation of the strain, bimolecular assay, and histological pattern were reported in order to provide a complete picture as the ovine epididymitis spread in a flock. We found a high seroprevalence in the tested rams (62.5% and 51.8% by ELISA and CFT assays respectively) in contrast to the lower prevalence detected in the ewes (3.5% and 4.9% by ELISA and CFT respectively). In our experience, differently, than other case reports [13,14,15], a high percentage (66.67%) of seropositive rams showed clinical signs and typical lesions.

**Figure 4:**
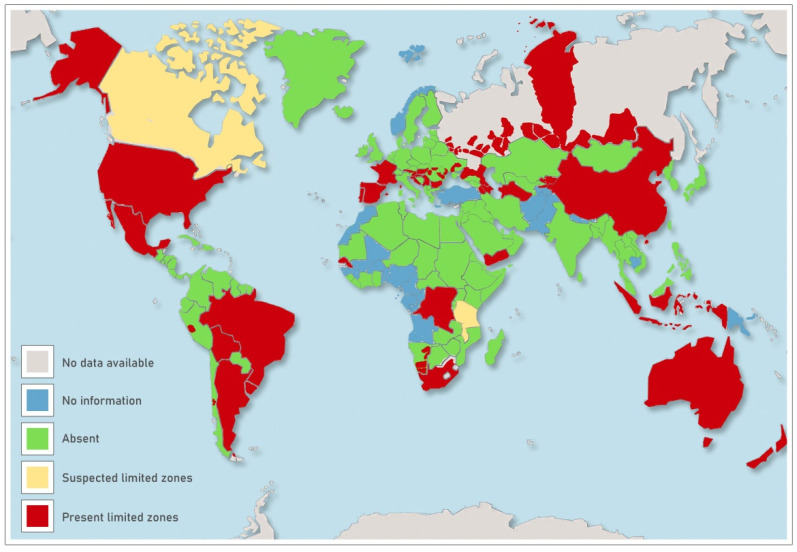
Countries reporting *Brucella ovis* infection worldwide.

To date, *B. ovis* infection in Sicily, as well as in Italy, is not well investigated and only a preliminary study on the spread of infection conducted on 942 rams belonging to 163 Sicilian farms reported a seroprevalence of 10% at the animal level [16]. In Italy, the disease was described for the first time in two rams in Lombardia in 1994 [15]. In relation to the reports drawn up by the OIE from 2013 to date this disease has never been notified officially in Italy (Figure 4).

Nevertheless, the isolation of *B. ovis* was also reported by local clinicians from sheep in the Trentino Alto-Adige, Campania, Marche [17,18], Piedmont, Abruzzi, and Lazio regions suggesting a significant presence of the infection in the Italian sheep population [12].

Different approaches for surveillance of the disease is reported from America, Asia, and Australia: data on seroprevalence were published in the livestock population of Wyoming [18] (0.53%), Idaho and Oregon (14.9%), Utah (27.1%), New Mexico (67.5%) and 10.0% in rams across Colorado, Wyoming, and Utah (10%) [19,20]. In Brazil, *B. ovis* seroprevalence ranging from 0.72% up to 2.89% [21,22]. In Europe, around 10% of seroprevalent animals were reported in New South Wales [5] and in the Basque Country in Spain [23], while a higher percentage was described in Serbia (29.8%) [24] and France (53.7% and 37.2% by ELISA and CFT assays respectively [25].

*B. ovis* natural infection has never been reported in wildlife, thus, the prevalence in other species is considered not relevant [26].

Sergeant [5], noted that the flock prevalence (percentage of flocks containing seropositive rams) was influenced by breed (9.1% for Merino flocks that were significantly lower than British-breed flocks 43.8%, and mixed-breed flocks 46.7%).

We reported a severe outbreak in the Sicilian Valle del Belìce dairy sheep breed confirming that the susceptibility to infection may vary among breeds of sheep. A study identified risk factors for ovine epididymitis to be the average age of rams in flocks, farms larger than 5 km^2^, and the lack of lambing paddocks [22].

Frequent isolation of *B. ovis* from the urine of slaughtered animals, from placentomes (cotyledon plus caruncle), and abomasal fluid confirms that in natural infection the microorganism may contaminate the environment through several biological fluids as well as proves that venereal route is not the only way of transmission [27]. Our molecular biology data were in agreement with these observations. In fact, in addition to identifying samples of genital swabs by real-time PCR, the specific DNA fragment for *B. ovis* was also detected in milk and urine samples. In particular, we found *B. ovis* DNA in 40% and 61.1% of milk and urine samples, respectively. Unfortunately, the pathogen has not been isolated probably due to the complexity of these matrices, which although widely used in diagnosis is very rich in fats. Although a selective medium was used, the long time needed for the isolation (six weeks) of this pathogen, often favors the growth of other microorganisms (especially molds) and makes the *Brucella* spp. isolation difficult.

Our positive PCR data on milk samples are in agreement with what was reported by Grillò et al. in 1999 [28]. Furthermore, those authors reported that in three sheep, artificially infected with *B. ovis*, mammary infection lasted for the whole lactation season till the next one. The persistent excretion of *B. ovis* in milk, with the consequent risk of perinatal transmission to the unweaned lambs, with the development of latent infections, are believed to play an important role in maintaining infection in the flock even without the role of rams [4].

However the OIE manual considers CFT as the most effective test, in this study 2 rams were diagnosed negative for CFT assay, but they were positive for ELISA and PCR and had evident both macroscopic and histopathological lesions. These data highlight the great importance of the use and comparison of more than one diagnostic method in order to verify true positives.

Although *B. ovis* infection was described worldwide, its control is still based on farm management including vaccination with the attenuated *B. melitensis* strain Rev.1 vaccine (where permitted). In other countries test and culling are applied, but complete eradication is extremely difficult to achieve and there is no report of a successful eradication campaign.

Usually, culling of symptomatic and positive rams is the only control measure applied. However, breeder often decides not to cull the seropositive rams because they are genetic improver in their breed and carrier of scrapie resistant genotype ARR/ARR. For this reason, any eradication measure should consider the status of each sheep population to preserve genetic and biodiversity together with disease control [29]. Despite, the disease has never been reported in Belgium, Denmark, Finland, Greece, Ireland, Luxembourg, Portugal, Sweden, the Netherlands, and the UK [30], the disease is not included among those diseases where mandatory eradication is requested. Thanks to the introduction of Reg. 429/2016, in force since April 2021, and with the application of the Commission Implementing Regulation (EU) 2018/1882, ovine epididymitis will be officially recognized among the MS as a “moderate risk disease” and addressed to specific surveillance and control measures to prevent its spread among MS or its entry into the EU.

The new regulation does not concern any special requirements for internal movement of the flocks but obliges all Member States (MS) to organize veterinary surveillance and laboratory certification if rams are moved between MS.

## 4. Materials and Methods

### 4.1. Anamnestic Data

The outbreak was identified in a traditional sheep farm, all ewes belonged to Valle del Belìce breed. The farm was located in the province of Trapani (Sicily) a district that concerns above 480 farms and a total population of 89,096 heads (latest census date back 2020) (IZS Teramo-Anagrafe Zootecnica) (https://www.vetinfo.it, accessed on 10 December 2020). In this area, livestock is conducted in a semi-extensive and extensive farming system with common grazing.

The farm counted a total number of 900 heads, of which 58 rams. The owner asked for veterinary consultation due to the presence of unusual swelling of the scrotum in several rams. However, problems of decreased fertility, abortion, or significant neonatal mortality are not reported by the owner.

### 4.2. Serological Analysis

Blood samples without anti-coagulant were collected from 56 rams and 143 ewes into a 10mLvacuum tube. Samples were allowed for clotting then were centrifuged (1500 rpm for 10 min) to recover the sera which were stored at −20 °C until tested. All sera were analyzed by two different tests: Enzyme-Linked Immuno Assay (ELISA) and Complement Fixation Test (CFT).

ELISA test was performed using a commercial kit (IDEXX *Brucella ovis*) applied as suggested for surveillance screening and the assay was done following the manufacturer’s instructions. As recommended samples were considered positive when the SP (sample-to-positive) % was above or equal to 45, while samples with SP% under 45 were interpreted as negative.

Complement Fixation Test was carried out by utilizing the protocol published in the OIE Manual [9]. Sera giving a titer equivalent to 50 ICFTU/mL (International CFT units) or more were considered positive.

The sample size was calculated considering a minimum expected prevalence of 2% (EFSA), with a confidence level of 95%, according to Winepi software (http://www.winepi.net/, accessed on 10 December 2020).

To evaluate CFT and ELISA tests agreement the Cohen’s kappa was calculated using GraphPad calculator (https://www.graphpad.com, accessed on 10 December 2020).

### 4.3. Molecular Analysis

Simultaneously to serological investigations, preputial swabs from all rams were collected. In addition, vaginal swabs and milk samples from 15 lactating ewes were also sampled.

Genomic DNA was extracted from tissues and milk using the commercial kit (Purelink Genomic DNA, Invitrogen, Thermo Fisher Scientific, Waltham, MA, USA) following the tissue protocol provided by the manufacturer. Urine samples were centrifuged at 13,000 rpm for 30′, the precipitated pellet was resuspended in 200 μL of 1X PBS (Phosphate Buffered Saline), while genital swab samples were immersed shook in 200 μL of 1X PBS: in both cases, the bacterial culture protocol provided by the manufacturer was followed [11]. Real-time PCR was performed to amplify a unique genetic locus, BOV_A0504. Real-time amplification was carried out in a total reaction volume of 25 μL containing 10 μL SSo advanced Universal Probes Supermix (Bio-Rad, Hercules, CA, USA), 0.5 µL of each primer (10 mM) and TaqMan probe (10 mM), 2 μL TaqMan^®^ Exogenous Internal Positive Control mix and 0.5 μL IPC template (Applied Biosystems, Thermo Fisher Scientific, Waltham, MA, USA) and about 100 ng/μL of DNA. The real-time PCR was performed using the CFX96 Touch Real-Time PCR Detection System (Bio-Rad). The instrumented program included an initial denaturation of 3 min at 95 °C followed by 40 cycles of amplification that included a denaturation step at 95 °C for 10 s and an annealing/extension step at 60 °C for 30 s. All samples with a Ct less than 38 were considered positive for *B. ovis*. All runs included a positive sample of *B. ovis* (REO 156) provided by the Italian National Reference Center for Brucellosis and a negative (DNAase/RNAase free water) control.

### 4.4. Clinical Examination and Sampling

Following serological and biomolecular investigations on sera and swabs respectively, a clinical examination by testicular palpation was performed in all rams. Therefore, the owner was agreed to eliminate all animals simultaneously positive to serological and molecular tests and clinical examination in order to clear the farm from animals at risk.

Eighteen positive rams were regularly slaughtered in an authorized slaughterhouse and post mortem inspection of the reproductive tract was performed.

Samples of testicle and epididymis from the 18 rams were collected in order to perform microbiological, molecular, and histopathological analyzes. In addition, 18 urine samples for molecular analysis were also collected.

### 4.5. Histology-Hematoxylin & Eosin Staining

A portion of pathological tissue of epididymis, testicle, and related lymph nodes (0.5 × 2 × 4 cm) was fixed in 10% buffered formalin. Serial sections of paraffin-embedded tissues of 4 µm thickness were cut using a microtome and set on slides treated with silane (3-aminopropyl-trieossi-silane) in order to avoid detachment during staining. The preparations obtained were dried overnight in an oven at 37 °C. It was proceeded with dewaxing by xylene for 20 min. After a descending alcohol series (100°, 95°, 75°, and 50°), slides were washed in distilled water and then stained with hematoxylin and eosin. This was followed by the ascending scale of alcohols (50°, 75°, 95°, and 100°) and clarification in xylene. After this phase, the slides were mounted in acrylic mounting medium (Eukitt^®^, O. Kindler GmbH, Baden-Württemberg, Germany).

### 4.6. Immunohistochemistry (IHC)

Immunohistochemical studies were performed on formalin-fixed paraffin-embedded pathological tissue, where a flogistic infiltrate was observed (mainly epididymis). Serial sections (4 μm thick) on glass slides were washed in xylene and hydrated in decreasing concentrations of alcohol. For antigen retrieval, the slides were heated in sodium citrate solution (pH 6.0) at 96 °C for 20 min. Endogenous peroxidase activity was quenched with 3% hydrogen peroxide in methanol for 30 min. Then the slides were treated with 1% bovine serum albumin (BSA) for 30 min and incubated for 1h at room temperature in the presence of 0.1% BSA with polyclonal rabbit, antibody anti-*Brucella* spp. (Byorbit), diluted 1:200 in 0.01 M PBS. In the end, the sections were treated for 30 min with secondary biotinylated immunoglobulin anti-rabbit antibody (DAKO, LSAB Kit, K0690, Denmark). The sections were then incubated with a streptavidin-horseradish peroxidase conjugate for 1 h, followed by chromogen 3-3′ diaminobenzidine tetrahydrochloride for 1 min, and counterstained with Mayer’s hematoxylin. The specific primary antibody was replaced with PBS in tissue sections used as negative controls. The DAB reaction developed a brown precipitate, when positive. Images of stained slides were captured by Leica DMR microscope equipped with a Leica DFC 320 digital camera and analyzed using digital image analysis (Nikon NIS Br, Nikon Instruments Europe BV, Amsterdam, The Netherlands).

### 4.7. Bacteriological Analysis and B. ovis Identification

Testicles, epididymis, lymph nodes, and urine of the 18 slaughtered rams were collected to perform a microbiological examination in order to isolate *B. ovis*.

Urine samples were streaked directly onto *Brucella* agar (modified Farrell’s selective medium) plates, as well as 1 mL was inoculated into 9 mL of *Brucella* broth. Fragments of tissues from testes, epididymis and lymph nodes were homogenized in phosphate buffer (PBS) containing amphotericin B and 1 mL of homogenate was then transferred into 9 mL of *Brucella* broth. All samples were incubated at 37 °C ± 2 °C with 5–10% CO_2_. Twenty-five microliter of broth was sown on *Brucella* Agar every six days, for six weeks to monitor the eventual growth of the pathogen [8].

Colonies attributable to *Brucella* spp. grew in *Brucella* agar were subcultured onto blood agar plates for 24–48 h at 37 °C in a 10% CO_2_ atmosphere. Before further investigation, after 24–48 h, colonies were subcultured onto Brain Heart Infusion Agar (BHI). On isolated strains, Gram staining, growth in MacConkey agar, growth in CO_2_ atmosphere, oxidase and catalase tests, mobility, urease, and H_2_S production, glucose oxide fermentation, growth in presence of basic fuchsin and thionin at a final concentration of 50 μg/mL were performed [31].

An agglutination test with *Brucella* A and M anti-serum was carried out. The *Brucella* polyvalent and monospecific *Brucella* A and M antisera were supplied by the Food and Agriculture Organization/WHO Collaborating Centre for Research on Brucellosis (Veterinary Laboratories Agency, Weybridge, UK). A 4 McFarland suspension of each strain was used to perform phage characterization by *Brucella*-phage of the Tbilisi (Tb), Weybridge (Wb), and Izatnagar (Iz1) groups [31].

### 4.8. Brucella spp. Typing

Genomic DNA from each strain isolated was extracted using the commercial kit (Purelink Genomic DNA, Invitrogen, Thermo Fisher Scientific, Waltham, MA, USA) following the bacterial culture protocol provided by the manufacturer. Typing was performed with AMOS (Abortus, Melitensis, Ovis, Suis) PCR. AMOS-PCR is a multiplex PCR designed to detect the polymorphism arising from species-specific localization of the insertion sequence IS711 in the chromosome of the four species of *Brucella* [32,33]. Amplicons were checked after electrophoresis in a 1% agarose gel.

## 5. Conclusions

This study performed on a *B. ovis* infected herd showed the clinical and diagnostic profile of the disease. Furthermore, it demonstrates the importance of the synergistic use of different diagnostic approaches that can facilitate the identification of infection. In order to allow faster identification and removal of chronic shedders, each farm should be monitored by a combination of serological tests (I-ELISA and CFT), molecular biology assays (PCR, real-time PCR), and auxiliary tests (genital palpation and seed culture whenever possible). This approach should be carried out at each breeding season, as well as before the introduction of new rams in the flocks.

Therefore, the introduction of Reg. 429/2016 establishes a new approach for proper, shared, and homogeneous veterinary measures in the whole EU against this pathogen which will also concern clinical and diagnostic protocols effective to detect even subclinical infections.

## Data Availability

Data are contained within the article.
